# Evidence, Mechanisms, and Clinical Implications of Microplastics and Nanoplastics As Emerging Cardiovascular Risk Factors: A Narrative Review

**DOI:** 10.7759/cureus.85696

**Published:** 2025-06-10

**Authors:** Sachidananda Moorthy, Srinivasan Kesavan, Sreerenjini Bhaskaran, Gayatri Balasubramanian, Madhulika Ambala, Khyathi Krishna Gogineni, Elizabeth Caroline Palaparthi, Likhith Sai Kiran Rapeti, Vignesh Vivekanandan, Panneerselvam Periasamy

**Affiliations:** 1 Pharmacology, Konaseema Institute of Medical Sciences and Research Foundation, Amalapuram, IND; 2 General Medicine, KMCH Institute of Health Sciences and Research, Coimbatore, IND; 3 Obstetrics and Gynaecology, PSG College Of Nursing, Coimbatore, IND; 4 Anaesthesiology, Government Erode Medical College, Erode, IND; 5 Medicine, Kamineni Academy of Medical Sciences and Research Centre, Hyderabad, IND; 6 Stroke Medicine, Stepping Hill Hospital, Stockport, GBR; 7 Internal Medicine, Shasta Regional Medical Center - Prime Healthcare, Redding, USA; 8 Community Medicine, NRI Institute of Medical Sciences, Visakhapatnam, IND; 9 Internal Medicine, SRM Medical College Hospital and Research Centre, Trichy, IND; 10 Physiology, Government Erode Medical College, Erode, IND

**Keywords:** atherosclerosis, cardiovascular disease, endothelial dysfunction, environmental toxicology, inflammation, microplastics, nanoplastics

## Abstract

This review comprehensively studied how microplastics and nanoplastics (MNPs) may affect heart and blood vessel health (cardiovascular diseases (CVDs)). These particles are found in the environment and can enter the human body through food, air, and skin contact. MNPs can travel through the bloodstream and build up in organs, including the heart and arteries. Studies in animals and humans suggest that MNPs may cause inflammation, damage blood vessels, disturb the normal function of the heart, and increase the risk of heart attacks and strokes. Researchers have also found MNPs in human artery plaques and blood clots. Current evidence of MNP on CVD is not fully conclusive. However, more research is needed to fully understand how much of a risk they pose. This review highlights the current knowledge, the possible health risks, the gaps in research, and what should be done next to better detect and manage these MNPs for public health safety.

## Introduction and background

The beginning of synthetic plastics in the early 20th century led to their entry into the manufacturing of healthcare, food packaging, and countless other sectors. Today, plastics have become a crucial component of modern life. Global plastic production exceeded 400 million tons in 2022 and is expected to reach an estimated 25 billion metric tons by 2050 [1]. However, this widespread utility comes at a significant environmental and public health cost. A significant proportion of plastic waste is derived from disposable consumer goods and industrial by-products. These can further undergo fragmentation into microplastics (MPs) (<5 mm) and nanoplastics (NPs) (<100 nm), and occur through physical, chemical, and biological processes [2].

Over the past decade, microplastics and nanoplastics (collectively referred to as MNPs) have emerged as environmental pollutants of concern. Following their release into the environment, MNPs pervade terrestrial, aquatic, and atmospheric systems via ocean currents, wind dispersal, and land runoff. MNPs can be perceived in water, soil, air, and food. The traces of MNPs have been reported in natural water bodies, soil, air, and even in our food, from where humans are chronically exposed to them through ingestion, inhalation, and dermal contact. Once internalized, MNPs can cross biological barriers and gain systemic access. Recent analytical advancements have confirmed the presence of plastic particles in human blood, lungs, liver, and even cardiovascular tissues [3]. A 2022 landmark study reported the detection of MPs around 0.7 μm in human blood samples, triggering critical questions regarding their physiological/pathophysiological implications [4].

Cardiovascular diseases (CVDs) include coronary artery disease, heart failure, stroke, and hypertension. CVDs remain the leading cause of mortality worldwide, accounting for approximately 17.9 million deaths annually. In vitro and in vivo studies suggest that MNPs may elicit cardiovascular toxicity through several biological pathways. MNPs induce oxidative stress, immune dysregulation, mitochondrial dysfunction, and endothelial injury [5]. Reduce cardiac output, myocardial fibrosis, and alter heart rate variability seen in the zebrafish and rodent models when exposed to polystyrene nanoplastics (PS-NPs) [6]. MNPs were recently detected in atherosclerotic carotid plaques, and their presence was associated with major adverse cardiovascular events such as myocardial infarction, stroke, and cardiovascular-related death in humans [7-9]. Animal studies propose that vascular deposition of MNPs could increase total peripheral resistance, promote platelet activation, and injure vascular endothelium in the case of endothelial dysfunction. Microvascular damage and predisposing individuals to ischemic events may be because of interactions between MNPs and blood cells [10].

A pivotal 2024 study was conducted using high-resolution pyrolysis-gas chromatography-mass spectrometry. It revealed that MNPs were detectable in excised human atherosclerotic plaques. This revealed that MNPs can infiltrate and accumulate within vascular lesions. This breakthrough not only substantiates prior preclinical data but also provides a framework for evaluating the role of MNPs in CVD pathogenesis. The current research highlights the need to clarify the cardiovascular relevance of MNPs and their persistent and continued bioaccumulation in human tissues. Yet, major knowledge gaps remain. These include insufficient longitudinal data, lack of standardized methodologies for MNP detection, limited toxicokinetic profiling, and absence of consensus on safe exposure thresholds [1-6].

Given this context, our review intended to evaluate and synthesize existing evidence on the presence, toxicological effects, and cardiovascular consequences of MNPs in humans, including mechanisms, tissue detection, clinical outcomes, research gaps, and future directions This narrative review is also focus on raising awareness about the potential health threats posed by MNPs and lay the groundwork for targeted investigations, regulatory action, and evidence-based clinical guidance.

## Review

Database search and selection

In accordance with the objective of this narrative review, the following search keywords were used to extract the literature databases (studies published between 2000 and 2025 were included) from PubMed, Scopus, Web of Science, and Google Scholar. The key search words or phrases are ("microplastics" OR "nanoplastics" OR "MNPs") AND ("cardiovascular disease" OR "atherosclerosis" OR "myocardial infarction" OR "stroke" OR "heart failure" OR "hypertension" OR "blood pressure") AND ("oxidative stress" OR "inflammation" OR "autophagy" OR "endothelial dysfunction" OR "senescence" OR "cardiotoxicity" OR "vascular damage" OR "foam cells" OR "platelet activation" OR "thrombosis" OR "clotting") AND ("detection" OR "accumulation" OR "tissue distribution" OR "blood" OR "plaque" OR "cardiac tissue" OR "vascular tissue") AND ("clinical outcomes" OR "mortality" OR "risk assessment" OR "epidemiology" OR "cohort study" OR "biomonitoring") AND ("future directions" OR "research gaps" OR "public health" OR "exposure limits").

Peer-reviewed articles, preclinical studies (in vivo, in vitro), human clinical, epidemiological studies, and systematic and narrative reviews were selected to extract the relevant data for this narrative review. Focused on relevance to MNPs, cardiovascular health, mechanistic pathways, and clinical outcomes. Articles were screened as per the inclusion criteria, and data were carefully synthesized as per the objective of this study. 

Pathophysiological mechanisms: how MNPs contribute to cardiovascular dysfunction (animal models and humans)

MNPs are emerging environmental contaminants with alarming implications for human health. Mounting evidence shows that MNPs can act as chronic disruptors of cardiovascular homeostasis through a constellation of molecular and cellular pathways. The complex nature of their toxicological actions spans immune modulation, oxidative stress, endothelial dysfunction, and thrombotic alterations. Each of these plays a central role in the pathogenesis of CVDs.

Entry, Systemic Distribution, and Cellular Uptake

MNPs gain entry into the human body primarily via ingestion, inhalation, and, to a lesser extent, via dermal contact. Contaminated food and water, urban air, textiles, personal care products, and household dust are the primary ways of entering the body [11]. Nanoplastics, particularly those <1 μm, translocate across epithelial barriers (gastrointestinal and pulmonary) via multiple molecular pathways. These include paracellular transport through tight junction disruption, Clathrin-mediated and caveolin-dependent endocytosis, and immune-cell-mediated transcytosis. Clathrin-coated pits facilitate the invagination of plasma membranes for nanoparticle internalization, while caveolae cholesterol-rich microdomains mediate transcytosis into deeper tissue layers. Additionally, MNPs can be recognized by pattern recognition receptors (e.g., toll-like receptor (TLR)2/4), leading to cytoskeletal rearrangements and vesicle trafficking involving Rab GTPases and endosomal sorting complexes. These mechanisms enable MNPs to bypass mucosal barriers and gain systemic access to vascularized tissues. These particles enter the bloodstream and further the lymphatic system, reaching vascularized tissues such as the heart, liver, kidneys, and atherosclerotic plaques. Detection of MNPs in human thrombi, carotid plaques, and even myocardial and pericardial tissues confirms their bio-accumulative potential [12-14].

Oxidative Stress and Mitochondrial Damage

One of the MNP-induced toxicities is reactive oxygen species (ROS) generation. After internalization at endothelial cells, macrophages, or cardiomyocytes, MNPs activate membrane-bound nicotinamide adenine dinucleotide phosphate (NADPH) oxidase complexes (particularly NADPH oxidase 2 (NOX2) and NADPH oxidase 4 (NOX4)), which catalyse the production of superoxide anions (O₂⁻). These reactive species impair mitochondrial respiration by disrupting electron transport at complexes I and III, leading to further ROS generation, mitochondrial membrane depolarization, and opening of the mitochondrial permeability transition pore (mPTP). This initiates cytochrome c release, caspase-9 activation, and downstream apoptotic signaling. Such a cascade culminates in oxidative damage to mitochondrial DNA, endothelial cell apoptosis, and vascular dysfunction, hallmarks of CVD pathogenesis [15,16]. Thus, this process leads to oxidative damage to mitochondrial DNA, disruption of the electron transport chain, and loss of ATP production. Endothelial damage and cellular apoptosis, thereby compromising vascular integrity, are promoted by Redox imbalance. These effects reflect classic oxidative mechanisms underlying hypertension, atherosclerosis, and ischemic heart disease [17,18].

Inflammation and Inflammasome Activation

MNPs activate pro-inflammatory cascades through multiple innate immune receptors, like TLR2 and TLR4, and the NOD-like receptor (NLR) family pyrin domain containing (NLRP3) inflammasome. Activation of nuclear factor kappa-light-chain-enhancer of activated B cells (NF-κB) and mitogen-activated protein kinase (MAPK) signaling that upregulates these cytokines: interleukin-1 beta (IL-1β), IL-6, tumor necrosis factor alpha (TNF-α), and intercellular adhesion molecule 1 (ICAM-1), establishing a persistent inflammatory milieu within vascular and cardiac tissues. MNPs positive plaques in humans demonstrate higher expression of cluster of differentiation 68 (CD68+) macrophages and CD3+ T-lymphocytes, supporting the clinical relevance of these pathways. The chronic inflammatory state also contributes to endothelial dysfunction, lipid uptake by macrophages, and fibrotic remodeling [18-21].

Endothelial Dysfunction

Endothelial cells are particularly vulnerable to MNP exposure due to the disruption of adherens junctions via VE-cadherin destabilization, phosphorylation (Y658, Y731), and cytoskeletal remodeling induced by nanoplastics, as demonstrated in both human endothelial cell cultures and animal vascular models [22]. MNPs induce endothelial leakiness, mitochondrial injury, and loss of nitric oxide (NO) bioavailability by downregulating endothelial NO synthase (eNOS) [22]. It might disrupt and then lead to vasodilation and increased vascular tone. Furthermore, MNPs promote the expression of adhesion molecules (vascular cell adhesion molecule-1 (VCAM-1), intercellular adhesion molecule-1 (ICAM-1)), facilitating leukocyte adherence and transmigration. Therefore, it is a key step in early atherogenesis. Endothelial aging triggered by redox-sensitive and proinflammatory signals further exacerbates vascular stiffness and impairs angiogenesis [23].

Atherogenesis and Plaque Instability

MNPs promote multiple pro-atherogenic processes. First, MNPs enhance macrophage uptake of oxidized-low-density lipoprotein (ox-LDL) by upregulating scavenger receptors such as CD36 and scavenger receptor (SR)-A1, likely via TLR4/NLRP3 inflammasome activation, leading to foam cell formation, a hallmark of early atheroma development [18]. Second, balance toward plaque growth and necrotic core development by altering lipid metabolism and increasing oxidized lipid species. Chronic inflammation, apoptosis, and pyroptosis of vascular cells lead to unstable plaques prone to rupture. The clinical relevance is underscored by findings from Marfella et al., who reported the presence of polyethylene (PE) and polyvinyl chloride (PVC) particles in over half of the examined human plaques, with associated increases in myocardial infarction, stroke, and mortality risk [24,25].

Cardiac Tissue Injury and Remodeling

MNPs disrupt intracellular calcium handling, mitochondrial respiration, and sarcomere architecture in cardiomyocytes through multiple mechanistic pathways. They impair calcium cycling by altering the expression and function of sarcoplasmic/endoplasmic reticulum Ca²⁺ ATPase (SERCA) and ryanodine receptors, leading to cytosolic calcium overload. Additionally, MNPs depolarize mitochondrial membrane potential and reduce ATP production, contributing to contractile dysfunction. Furthermore, activation of the Wnt/β-catenin and TGF-β1/small mothers against decapentaplegic proteins (Smad) signaling pathways has been observed in MNP-exposed myocardium, promoting fibrotic remodeling and cardiomyocyte hypertrophy. These effects reduce contractility, alter β-adrenergic signaling, and may promote cardiac arrhythmias. MNPs exposure in animal models results in bradycardia, pericardial oedema, and hypertrophy [26]. A pathway involving Wnt proteins and β-catenin has been mechanistically linked to MNP exposure, particularly PS-NPs, which have been shown to upregulate Wnt target genes and promote β-catenin accumulation in zebrafish cardiac tissues. This aberrant activation contributes to cardiac remodeling and fibrosis. Concurrently, transforming growth factor beta 1 (TGF-β1)/Smad signaling is also triggered, further amplifying fibrotic and hypertrophic changes [27]. Biomarkers, including troponin-I and creatine kinase-MB (CK-MB), are significantly elevated following MNP exposure. For instance, in rat models exposed to polystyrene (PS) microplastics, serum troponin I levels increased by 62.4% and CK-MB levels by 48.7% compared to control animals, indicating subclinical myocardial injury [26].

Hematological Disruption and Thrombosis

Recent evidence elucidates how MNPs, particularly PS-NPs, interact detrimentally with erythrocytes. These particles induce oxidative stress, disrupting the integrity of the red blood cell membrane by altering lipid bilayer thickness and curvature, ultimately impairing cell deformability. Functionalized PSNPs (e.g., amine-modified) exhibit heightened hemolytic activity by promoting phosphatidylserine externalization and micro-vesicle formation. Additionally, PSNPs have been shown to bind human hemoglobin at the hydrophobic β-chain pocket, potentially altering its conformation and impairing oxygen transport. In vivo models demonstrate poikilocytosis, eryptosis, and red cell fragility upon MNP exposure, underscoring their contribution to hemolysis, impaired oxygen delivery, and increased thrombotic risk. This has been corroborated by histological detection of MNPs in human arterial and venous thrombi. The net effect is a heightened thrombotic risk, particularly in low shear venous environments or inflamed vessels [28].

Autophagy Impairment and Cell Death

MNPs block autophagic flux in endothelial and myocardial cells. Such incidents lead to the accumulation of damaged mitochondria and proteins, ultimately promoting apoptosis or pyroptosis. Pyroptosis, mediated via the NLR family pyrin domain containing 3 (NLRP3)/Caspase-1/Gasdermin D (GSDMD) axis, contributes to sterile inflammation within vascular walls and myocardium, also a central feature of CVD pathology [29,30].

Gut-Immune-Cardiovascular Axis

Emerging evidence highlights the gut-immune-vascular axis as an important link in MNP-induced cardiotoxicity. MNPs alter gut microbiota composition, increase intestinal permeability, and then trigger systemic immune dysregulation. Such alterations promote low-grade inflammation and metabolic endotoxemia, which are accelerants of atherosclerosis and hypertension. Additionally, gut-primed Regulatory T cells (T-reg) and T helper type 17 cells (Th17) imbalances may influence peripheral vascular inflammation and immune responses [31,32].

Endocrine and Metabolic Modulation

Many MNPs carry adsorbed endocrine-disrupting chemicals (EDCs), including bisphenol A (BPA), phthalates, and PBDEs, which interfere with the hormonal regulation of metabolism and cardiovascular risk. These EDCs are not covalently bound and readily leach into biological fluids, disrupting multiple endocrine axes. Evidence shows that MPs and NPs can alter thyroid hormone levels (e.g., decreasing T3, FT3, FT4, and increasing TSH) and impair hypothalamic-pituitary-thyroid (HPT) function in animal models. For instance, PS-NPs at 1-10 mg/kg/day reduced circulating thyroid hormones in rats, while phthalates altered gene expression in the HPG axis and promoted anti-androgenic effects. BPA disrupts steroidogenesis, reduces testosterone, and increases plasma corticosterone through SHH pathway activation. Additionally, MNPs bioaccumulate and act as "Trojan Horses," transporting toxicants that dysregulate insulin signaling, elevate corticosterone, and promote metabolic disorders, including obesity, insulin resistance, and dyslipidemia - all established cardiovascular risk factors [33].

Conceptual Integration

MNPs with complex interrelated mechanisms contribute to cardiovascular dysfunction. The pathophysiological sequence begins with systemic entry and bioaccumulation, followed by cellular stress, inflammatory activation, endothelial dysfunction, and lipid disruption. These changes culminate in structural vascular alterations, thrombo-inflammation, and myocardial injury. Particularly, these mechanisms line up closely with those implicated in traditional cardiovascular risk pathways. It has been suggested that MNPs may act as novel, modifiable, and environmentally driven contributors to CVD burden, and this is summarized in Table 1.

**Table 1:** Summary of Pathophysiological Mechanisms Linking MNPs to Cardiovascular Disease This table summarizes the primary biological mechanisms by which microplastics and nanoplastics (MNPs) are hypothesized to induce cardiovascular dysfunction. ATP, adenosine triphosphate; BPA, bisphenol A; CD36, cluster of differentiation 36; EDCs, endocrine-disrupting chemicals; HPG axis, hypothalamic-pituitary-gonadal axis; HPT axis, hypothalamic-pituitary-thyroid axis; IL-1β, interleukin 1 beta; LDL, low-density lipoprotein; MNPs, microplastics and nanoplastics; NF-κB, nuclear factor kappa B; NLRP3, NOD-, LRR- and pyrin domain-containing protein 3; NO, nitric oxide; NOX2/NOX4, NADPH oxidase isoforms 2 and 4; ROS, reactive oxygen species; RyR, ryanodine receptor; SERCA, sarco/endoplasmic reticulum Ca²⁺-ATPase; Smad, small mothers against decapentaplegic proteins in TGF-β signaling; SR-A1, scavenger receptor class A type 1; T3 and T4, triiodothyronine and thyroxine; TGF-β1, transforming growth factor beta 1; Th17/Treg, T-helper 17 cells and regulatory T cells; TLR2/4, toll-like receptor 2 and 4; TNF-α, tumor necrosis factor alpha; VE-cadherin, vascular endothelial cadherin; β-adrenergic, beta-adrenergic receptors

Pathophysiological Effects	Mechanism
Systemic entry and distribution [11,17,34]	MNPs enter the body via ingestion, inhalation, and dermal exposure. Nanoplastics (<1 µm) cross epithelial barriers through paracellular transport, Clathrin-mediated endocytosis, and immune cell-mediated transcytosis. They accumulate in blood, atherosclerotic plaques, and cardiovascular tissues due to limited clearance and high tissue retention.
Oxidative stress [17,18]	MNPs activate NADPH oxidases (NOX2/NOX4), generating ROS, which impairs mitochondrial electron transport (complexes I and III), leading to mitochondrial membrane depolarization, cytochrome c release, DNA damage, and ATP depletion.
Inflammation and immune activation [18-21]	Surface properties of MNPs trigger toll-like receptors (TLR2/4) and activate NF-κB and NLRP3 inflammasome pathways. This results in increased IL-1β, TNF-α, and recruitment of neutrophils and macrophages, contributing to chronic low-grade vascular inflammation.
Endothelial dysfunction [23]	MNPs disrupt endothelial tight junctions via VE-cadherin phosphorylation (Y658, Y731), reduce nitric oxide (NO) bioavailability, and induce mitochondrial damage. This compromises endothelial barrier integrity, increases vascular permeability, and promotes endothelial apoptosis.
Atherogenesis and plaque instability [24,25]	MNPs enhance macrophage uptake of oxidized-LDL via upregulation of scavenger receptors (CD36, SR-A1), promoting foam cell formation. This, combined with lipid dysregulation and macrophage apoptosis, accelerates necrotic core development and plaque vulnerability.
Cardiac injury and fibrosis [26]	In cardiac tissues, MNPs impair β-adrenergic signaling, disrupt calcium handling (SERCA, RyR), and induce mitochondrial dysfunction. Activation of Wnt/β-catenin and TGF-β1/Smad pathways promotes cardiomyocyte apoptosis, hypertrophy, and fibrotic remodeling.
Thrombosis and coagulation disruption [28]	MNPs interact with erythrocyte membranes, causing hemolysis, and enhance platelet adhesion and aggregation via surface charge effects and ROS generation. This initiates fibrin polymerization and thrombus formation, especially under low shear conditions.
Autophagy impairment [29,30]	MNPs inhibit autophagic flux by disrupting lysosomal integrity and autophagosome-lysosome fusion. This leads to the accumulation of damaged organelles, oxidative stress, and activation of pyroptosis (via caspase-1) and apoptosis pathways.
Gut-immune-cardiovascular axis dysregulation [31,32]	MNPs alter gut microbiota composition, increase intestinal permeability (leaky gut), and promote systemic endotoxemia. This contributes to Th17/Treg imbalance, vascular inflammation, and metabolic endotoxemia-linked endothelial dysfunction.
Endocrine disruption and metabolic effects [33]	MNPs serve as carriers for EDCs like bisphenol A (BPA) and phthalates, which disrupt the HPT and HPG axes. They alter hormone receptor activity, reduce thyroid hormone (T3, T4) levels, impair insulin signaling, promote adipogenesis, and induce hypertension and dyslipidemia.

Clinical outcomes and epidemiological signals: MNP presence to real-world CVD outcomes

The pathophysiological data have laid the foundation for concern. A substantial shift in recent research has been the emergence of clinical and epidemiological evidence linking MNP exposure to CVD outcomes in humans. This section synthesizes insights from the following foundational studies to assess the real-world cardiovascular implications of MNP accumulation.

Prospective Human Evidence: MNPs and Major Cardiovascular Events

Marfella et al. identified MNPs in 58.4% of excised plaques, with a marked increase in myocardial infarction, stroke, and mortality among MNP-positive individuals. The adjusted hazard ratio for the composite outcome was 4.53 (95% CI: 2.00-10.27), signifying more than a fourfold increase in event risk over ~34 months of follow-up. These findings were supported by elevated cytokine levels (IL-6, IL-1β, TNF-α, IL-18) and immune cell infiltration (CD68+, CD3+), also indicating a proinflammatory, vulnerable plaque phenotype associated with MNP burden [24].

Clinical Correlates of MNPs in Atherosclerosis and Thrombosis

Multiple independent reviews validate MNP enrichment in arterial plaques and thrombi from patients with ischemic cardiovascular conditions. For example, Yang et al. found MNPs in 100% of acute coronary syndrome (ACS) patients’ blood samples, with positive correlations between MNP load and Synergy Between PCI With Taxus and Cardiac Surgery (SYNTAX) scores, a measure of coronary lesion complexity [35]. Thrombi retrieved from patients with stroke, myocardial infarction, or deep vein thrombosis revealed MNP presence in up to 80% of cases. Further, concentrations of MNPs correlate with biomarkers such as D-dimer and platelet counts. These findings stress a link between MNPs and both the thrombotic burden and complexity of vascular disease [36].

Impact on Cardiovascular Biomarkers and Risk Indicators

In some non-CVDs, the MNP exposure has been associated with surrogate markers of vascular pathology. Endothelial cells exposed to MNPs exhibit impaired autophagic flux, increased agedness, and upregulation of adhesion molecules (ICAM-1, VCAM-1), all of which are indicative of early endothelial dysfunction, a known precursor of atherosclerosis. Furthermore, a pilot human study demonstrated that reduced plastic ingestion correlated with decreased blood pressure. Thus, suggesting a potential modulatory role of MNP exposure in hypertension development, a key cardiovascular risk factor [32].

Immune Activation, Chronic Inflammation, and Plaque Instability

Across studies, MNPs have been shown to activate macrophages and T-cells, promoting a milieu of chronic inflammation through the release of IL-1β, IL-6, and TNF-α. These immune responses mirror those observed in vulnerable atherosclerotic plaques and are consistent with the pro-inflammatory environment that facilitates plaque rupture and acute vascular events [37]. The reduction in plaque collagen content and increased infiltration of inflammatory cells in MNP-positive cases provide further histopathological validation [27].

In summary (some of the MNPs studies on humans are summarized in Table 2), MNPs are bioavailable, bio-accumulative, and biologically active in human CVS. Detection in atherosclerotic plaques, thrombi, and systemic circulation, coupled with immune activation and endothelial dysfunction. This evidence provides a mechanistic link to CVD. Critically, prospective data now demonstrate a robust association between MNP-positive vascular tissues and adverse cardiovascular outcomes, including myocardial infarction, stroke, and mortality.

**Table 2:** Summary of Key Human Studies Investigating MNPs in Cardiovascular Tissues and Outcomes This table compiles major clinical and epidemiological studies that have detected MNPs in human cardiovascular samples or associated MNP burden with adverse cardiovascular outcomes. ACS, acute coronary syndrome; CD3⁺, cluster of differentiation 3-positive (T-cell marker); CD68⁺, cluster of differentiation 68-positive (macrophage marker); D-dimer, fibrin degradation product (marker of thrombotic activity); DVT, deep vein thrombosis; GC/MS, gas chromatography-mass spectrometry; IL-6, interleukin-6; MI, myocardial infarction; MNPs, microplastics and nanoplastics; PA66, polyamide 66 (nylon 66); PE, polyethylene; PET, polyethylene terephthalate; PP, polypropylene; PS, polystyrene; PVC, polyvinyl chloride; SYNTAX score, synergy between PCI with Taxus and cardiac surgery score (coronary lesion complexity index); TNF, tumor necrosis factor

Authors With Year	Study Name	Sample Analyzed	Technique Used	MNPs Detected	Findings	Outcomes
Marfella et al. (2024) [24]	Prospective carotid endarterectomy study	Carotid plaques from 257 patients	Electron microscopy, isotope analysis	PE, PVC	MNPs in 58.4% of plaques; increased cytokines (IL-6, TNF), CD68+/CD3+ cells, plaque instability	4.53× higher risk of MI, stroke, and death in MNP-positive patients
Yang et al. (2024) [35]	ACS study	Blood from ACS patients	Spectroscopy	Various MNPs	100% of ACS patients had MNPs; correlated with SYNTAX score	Greater coronary lesion complexity
Wang et al. (2024) [36]	Thrombi and cardiovascular event study	Thrombi from MI, stroke, DVT patients	Raman spectroscopy	PE, PA66, PVC	MNPs found in up to 80% of thrombi; associated with elevated D-dimer	Prothrombotic role of MNPs
Zhang et al. (2025) [27]	MNPs in patients with MI	Cardiovascular tissues (saphenous veins, myocardium, etc.)	Spectroscopy, pyrolysis-GC/MS	PVC, PE, PET	MNPs in multiple tissues; associated with inflammation	Supportive of MNP role in atherosclerosis and thrombosis
Liu et al. (2024) [38]	Atherosclerotic vs. non-atherosclerotic arteries	Arterial tissue (human surgery samples)	Chemical and histological analysis	PET, PE	Higher MNP load in diseased arteries	Preferential accumulation in pathological vessels
Yan et al. (2023) [39]	Fecal MNPs and vascular calcification study	Fecal samples from 47 individuals	Spectroscopy	PP, PS	Higher fecal MNPs in patients with aortic calcification	Indirect link to early vascular damage

Detection methods and plastic characteristics: challenges and inconsistencies

The detection and characterization of MNPs constitute one of the most critical methodological bottlenecks in advancing our understanding of their biological and cardiovascular impacts. This section synthesizes current literature to highlight key challenges in MNP detection and plastic characterization across experimental and clinical settings.

Ambiguous Size Classifications and Polymer Diversity

The detection dilemma lies in a lack of standardized definitions. While microplastics are broadly defined as particles ranging from 1 μm to 5 mm, nanoplastics are variously classified as <1 μm or <100 nm depending on the regulatory or research context. This taxonomic ambiguity results in variable inclusion criteria, complicating epidemiological synthesis and regulatory assessment. Polymer diversity further adds complexity. Commonly detected polymers in human tissues include PE, polypropylene (PP), PS, PVC, polyethylene terephthalate (PET), and polyamide. However, the dominance of PS in experimental models driven by its commercial availability does not reflect real-world exposure, where PE and PET are more prevalent. This misalignment introduces significant bias in toxicity data and limits ecological and physiological relevance [40].

Morphological and Surface Complexity

MNPs exhibit significant variability in morphology (spherical, fibrous, jagged) and surface chemistry (e.g., aminated, carboxylated, weathered). Such properties govern particle behavior, cellular uptake, and toxicological potential. For example, irregularly shaped PVC particles elicit stronger immune activation compared to smoother counterparts, while surface modifications such as amino groups enhance endothelial toxicity. The particle shape and surface charge are inconsistently characterized or entirely omitted in many studies. This omission restricts mechanistic interpretation and inter-study comparability, particularly in studies examining cardiovascular tissues [41].

Detection in Biological Matrices: Analytical and Biological Barriers

Detecting MNPs in biological matrices such as blood, tissue, or plaque presents a unique challenge. Biological material is prone to optical and chemical noise, complicating spectroscopy-based identification. Protein corona formation: a ubiquitous phenomenon in vivo, and such obstruction can obscure surface signals or alter particle interactions, reducing detection clarity. Furthermore, contamination is an omnipresent risk. Airborne plastic particles, polymer-based labware, and procedural cross-contamination can introduce false positives, particularly in low-burden samples such as arterial tissue or serum. Yet few studies incorporate rigorous contamination control (e.g., procedural blanks, clean-room conditions), compromising data reliability [42,43].

Quantification Challenges and Unit Inconsistencies

A persistent limitation in the MNP literature is inconsistency in quantification metrics. Studies variably report particle count per volume (particles/mL), mass concentration (μg/g), or surface area. Some reports only report particle presence or absence. This heterogeneity obstructs inter-study comparisons, meta-analyses, and dose-response modelling. Moreover, particle aggregation and physicochemical transformations during sampling (e.g., swelling in solvents) can distort measured size distributions, leading to misclassification, particularly of nanoplastics as microplastics [42,43].

Real-World vs. Experimental Mismatch

A striking disparity exists between particles detected in real human samples and those used in experimental models. Environmental MNPs are weathered, chemically modified, and complex in composition, whereas lab-prepared particles are often original, monodisperse, and polymer-specific (mostly PS). Such models, often applied at supra-environmental concentrations, fail to replicate actual human exposure, diminishing translational value. For instance, Marfella et al. detected only PE and PVC in carotid artery plaques, despite screening for 11 polymers. This may reflect detection bias, tissue retention preferences, or both. Regardless, it emphasizes the need for environmentally relevant materials in toxicity testing. The current landscape of MNP detection and characterization is marred by substantial inconsistencies in definitions, methodologies, and reporting [42,43].

Limitations

Despite the convincing body of evidence linking MNPs to cardiovascular toxicity, the current state of research remains constrained by several critical limitations that undermine conclusive risk assessments and translational applicability. These limitations span methodological, epidemiological, toxicological, and regulatory domains, underscoring the need for targeted refinements in study design, technology, and interdisciplinary collaboration. Limitations broadly include: methodological heterogeneity and detection challenges, limitations in human exposure assessment, sparse and preliminary epidemiological evidence, mechanistic ambiguities and translational gaps, and lack of regulatory and clinical integration [27,38-44].

Future directions

To effectively mitigate the cardiovascular risks associated with MNPs, several strategic research priorities must be pursued. First, standardized definitions and validated detection protocols are urgently needed to ensure consistency across studies, particularly in biological matrices such as blood, plaques, and cardiac tissue. Experimental models should evolve to reflect real-world conditions by incorporating environmentally weathered, heterogeneous MNPs with varied polymer types and adsorbed contaminants. Prospective cohort studies integrating MNP biomonitoring into existing biobanks and cardiovascular registries will be essential for establishing dose-response relationships and identifying at-risk subpopulations. Mechanistic insights should be advanced through multi-omics approaches and in vivo imaging techniques to map molecular and tissue-level effects, while the development of non-invasive exposure assessment tools will support surveillance and intervention strategies. Lastly, interdisciplinary, policy-linked research is crucial to inform regulatory action, define exposure thresholds, and protect vulnerable populations through evidence-based public health guidelines.

## Conclusions

MNPs are tiny plastic particles that have become an unexpected threat to heart and blood vessel health. This review brings together growing evidence from laboratory studies, animal experiments, and human research showing that these particles can enter the body through food, air, or skin, travel through the bloodstream, and build up in the heart, arteries, and other organs. Once inside, MNPs can damage cells, trigger inflammation, disrupt normal heart and blood vessel function, and increase the risk of clots. Studies have even found these plastics in human artery plaques, linking them to heart attacks, strokes, and higher death rates. They may also upset hormone balance and gut health, factors that play a role in blood pressure and heart disease, suggesting that MNPs could be a hidden, but preventable, contributor to CVD.

At the same time, there are still many things that remain unexplored. Current methods to detect MNPs in the body are inconsistent, and many laboratory studies use unrealistic types or amounts of plastics. We need better tools to measure real-life exposures, more long-term studies in humans, and better models that reflect everyday plastic exposure. Using modern technologies like genetic and protein analysis can help us learn how these particles affect the body at a deeper level. Finally, scientists, doctors, and policymakers need to work together to raise awareness, improve regulations, and develop ways to reduce our exposure to harmful plastic particles, especially in vulnerable populations such as individuals living near plastic production or waste incineration sites, workers in plastic manufacturing or recycling industries, socioeconomically disadvantaged communities with poor environmental quality, and biologically susceptible groups including children, the elderly, and those with pre-existing cardiovascular or metabolic diseases.
